# Climate Change Induces Shifts in Abundance and Activity Pattern of Bacteria and Archaea Catalyzing Major Transformation Steps in Nitrogen Turnover in a Soil from a Mid-European Beech Forest

**DOI:** 10.1371/journal.pone.0114278

**Published:** 2014-12-02

**Authors:** Silvia Gschwendtner, Javier Tejedor, Carolin Bimueller, Michael Dannenmann, Ingrid Kögel Knabner, Michael Schloter

**Affiliations:** 1 Research Unit Environmental Genomics, Helmholtz Zentrum München, German Research Center for Environmental Health (GmbH), Ingolstädter Landstraße 1, 85764, Neuherberg, Germany; 2 Institute of Meteorology and Climate Research, Atmospheric Environmental Research (IMK-IFU), Karlsruhe Institute of Technology (KIT), Kreuzeckbahnstrasse 19, 82467, Garmisch-Partenkirchen, Germany; 3 Lehrstuhl für Bodenkunde, Department of Ecology and Ecosystem Management, Center of Life and Food Sciences Weihenstephan, Technische Universität München, D-85350, Freising-Weihenstephan, Germany; North Carolina State University, United States of America

## Abstract

Ongoing climate change will lead to more extreme weather events, including severe drought periods and intense drying rewetting cycles. This will directly influence microbial nitrogen (N) turnover rates in soil by changing the water content and the oxygen partial pressure. Therefore, a space for time climate change experiment was conducted by transferring intact beech seedling-soil mesocosms from a northwest (NW) exposed site, representing today's climatic conditions, to a southwest (SW) exposed site, providing a model climate for future conditions with naturally occurring increased soil temperature (+0.8°C in average). In addition, severe drought and intense rainfall was simulated by a rainout shelter at SW and manual rewetting after 39 days drought, respectively. Soil samples were taken in June, at the end of the drought period (August), 24 and 72 hours after rewetting (August) and after a regeneration period of four weeks (September). To follow dynamics of bacterial and archaeal communities involved in N turnover, abundance and activity of nitrifiers, denitrifiers, N_2_-fixing microbes and N-mineralizers was analyzed based on marker genes and the related transcripts by qPCR from DNA and RNA directly extracted from soil. Abundance of the transcripts was reduced under climate change with most pronounced effects for denitrification. Our results revealed that already a transfer from NW to SW without further treatment resulted in decreased *cnor* and *nosZ* transcripts, encoding for nitric oxide reductase and nitrous oxide reductase, respectively, while *nirK* transcripts, encoding for nitrite reductase, remained unaffected. Severe drought additionally led to reduced *nirK* and *cnor* transcripts at SW. After rewetting, *nirK* transcripts increased rapidly at both sites, while *cnor* and *nosZ* transcripts increased only at NW. Our data indicate that the climate change influences activity pattern of microbial communities involved in denitrification processes to a different extend, which may impact emission rates of the greenhouse gas N_2_O.

## Introduction

Beech (*Fagus sylvatica* L.) dominates the natural forest vegetation from moderate dry to moist areas of sub-mountainous altitude in Central Europe [1]. It often grows on calcareous, limestone derived soils which have low water retention capacity and are poor in bioavailable nitrogen (N) [2], [3]. However, N is an essential component of proteins, nucleotides, coenzymes, photosynthetic pigments, secondary metabolites and other molecules, and is one of the major growth limiting factors for plants [4]. Consequently, productivity of forest ecosystems is strongly dependent on an efficient microbial N turnover characterized by low N losses via denitrification or leaching and rapid recycling of organically bound nutrients by mineralization [5]. Furthermore, in N-limited ecosystems strong competition between microorganisms and plants for N exists, leading to a fragile balance of microbial N mineralization and N immobilization by microbes and plants, which is determined mainly by abiotic factors, including soil temperature, oxygen partial pressure, pH and nitrogen and carbon (C) availability [6], [7]. Consequently, altered environmental conditions induced by global change may lead to a significant disturbance of unfertilized forest ecosystems. Mainly an increase in extreme weather events, including a higher frequency of intense precipitation and thus rapidly changing soil moisture regimes, extreme temperature events, heat waves and an increase in frequency and duration of drought periods [8] might affect the interplay of soil biota and plants in the future.

It is well accepted that drought decreases microbial activity and biomass due to osmotic regulation (accumulation of compatible solutes in cells to avoid dehydration), limited diffusive transport of substrates and extracellular enzymes and decreased microbial motility [9]. Furthermore, root biomass and consequently root exudates are reduced [10], resulting in additional C limitation for soil microorganisms. However, increased oxygen content due to lower soil moisture in combination with reduced competition for N due to reduced plant performance might lead to enhanced nitrification, which is an aerobic process performed by autotrophic bacteria and archaea independent from labile C sources [11]. Increased nitrification will lead to nitrate formation, which may leach to the ground water depending on soil type, reducing the available N pools in soil to a large extend.

Rewetting of dry soils induces additional osmotic stress for microbes, resulting in release of cytoplasmic solutes to avoid bursting [12]. However, microbial activity was shown to increase within minutes [13] or hours [14] after rewetting, mainly due to reconstituting mineralization of both newly exposed organic matter and dead microbial cells accumulated during drought [15]. This nutrient flush in combination with reduced oxygen levels due to high soil moisture leads to enhanced denitrification activities [16], [17] and thus increased N losses from the ecosystem. Nevertheless, the response of microbial processes to environmental stress conditions is not only related to soil moisture and the availability of nutrients and organic matter, but also to changes in microbial community structure [16], [17]. Recent studies on the dynamics of microbes involved in N turnover under different environmental stress factors provide contradictory results. When investigating the influence of temperature and water content on bacterial, archaeal and denitrifying microbial communities, Stres et al. [18] found no significant response of bacterial and denitrifier abundance and community structure, while archaeal communities were strongly influenced by temperature. In contrast, denitrifying communities in a pristine forest soil were highly affected by water content, and both archaeal ammonia oxidizers (AOA) and their bacterial counterpart (AOB) responded to different temperatures and water regimes [19]. These contradicting results indicate that the effects of climate change conditions on microbial communities and their functional traits are still poorly understood and cannot be easily transferred from one ecosystem to another.

Whereas most studies so far investigated either the effects of drought or intensive rainfall on soil microbial communities, data on extreme drought rewetting cycles are still rare, despite their relevance for future climate change scenarios [8]. Thus, in the frame of this project we performed a transplant experiment where intact beech seedling-soil mesocosms from a northwest (NW) exposed site, representing today's climatic conditions, were transferred to a site of southwest (SW) exposure, serving as model for changed climatic conditions. Additionally, a rainout shelter was established at SW to simulate severe drought. After 39 days drought, intense precipitation was simulated by manual rewetting of the mesocosms at both NW and SW site. Soil samples were taken in June (before the rainout shelter was established), at the end of the drought period (August), 24 and 72 hours after rewetting (August) and at the end of the beech vegetation period in September.

In contrast to previous studies investigating climate change effects on soil microflora focusing on shifts in microbial community structure [16], [17] or on effects on single N transformation steps [18], [19], in the present study for the first time consequences for activity pattern of N_2_-fixers, nitrifiers, denitrifiers and microbes involved in N-mineralization were investigated in addition to shifts in abundance of bacterial and archaeal communities involved in N cycling. Therefore, the abundance of marker genes of prokaryotes encoding for enzymes catalyzing key processes in N turnover as well as their transcripts were quantified using quantitative real-time PCR (qPCR) based on extracted DNA and mRNA from soil (N_2_ fixation (*nifH*), nitrification (*amoA*) and denitrification (*nirK, nirS, cnor* and *nosZ*)). The second known gene encoding for nitric oxide reductase, *qnor*, was not considered, as it was also found in many non-denitrifying microorganisms, suggesting a function in detoxifying NO [20]. From the large pool of genes encoding for N mineralization enzymes alkaline metalloprotease (*apr)* and chitinase (*chiA)* were chosen as marker genes in this study. In temperate forest ecosystems, most tree species form ectomycorrhizal fungal (EMF) associations, and especially beech roots are usually 100% colonized by EMF [21], resulting in a high soil chitin concentration. Proteins comprise up to 40% of the total soil N [22], and there is evidence that proteolysis could be the rate-limiting step in soil organic N mineralization [23]. Hence, the hydrolysis of proteins and chitin is particularly important to supply the soil N pool with plant available N. Furthermore, it was shown that the abundance of *chiA* and *apr* genes correlates positively with chitinolytic and proteolytic enzyme activities [24] and that both chitinase and protease activity correlates positively with N mineralization rate [23], [25].

## Materials and Methods

### Study site description

Permits for performing the experiment were issued by the Landratsamt Tuttlingen, Germany. The experiment was conducted at the Tuttlingen Research Station, a long term ecological beech research forest site in the Swabian Jura, a low mountain range in Southern Germany (8°45′E/47°59′N) at an altitude of 800 m above sea level with an atmospheric N deposition of <10 kg N ha^−1^ year^−1^ [26]. The soil was classified as Rendzic Leptosoil (Sceletic) according to the IUSS [27] with the following characteristics: 68% clay, 28% silt, 4% sand, pH 6.0 (measured in 0.01 M CaCl_2_), 0.5% total nitrogen content, 6.1% organic carbon content, 0.05% inorganic carbon content and a maximum water holding capacity (WHC) of 118% [28]. To simulate climate change, two sites with opposing exposure (NW and SW) of a narrow valley within a distance <1 km of each other were selected as model ecosystems. At both sites, beech was the dominant species and trees showed similar population genetics [29]. The NW site showed a cool-moist climate representing present climatic conditions typical of a beech stand in Central Europe with mean annual air temperature of 6.6°C, mean air temperature during growing season (April to October) of 11.5°C, mean annual precipitation of 810 mm and mean precipitation during growing season of 86 mm [30]. The SW site was characterized by higher radiation, resulting in increased daily maximum of air and soil temperature and thus reduced water availability [31], representing future climatic conditions [8]. During the experimental period, mean soil temperature/volumetric soil moisture at NW and SW were 10.3°C/0.26 m^3^ m^−3^ and 11.1°C/0.23 m^3^ m^−3^, respectively [28].

### Experimental design

This study was part of a transplant experiment investigating N turnover in European beech forests under climate change conditions. The transfer of the intact plant-soil mesocosms was described in detail by Bimueller et al. [28]. In brief, stainless steel cylinders (diameter 16.8 cm, height 15 cm) were used to sample 80 soil cores centered around a beech seedling of 2 mm stem diameter and 30 cm height without disturbing the root architecture. Sampling was done in July 2010 on a preselected sampling area on the NW site of the beech forest, where intensive drilling had revealed similar soil profiles with weathered bedrock occurring at > 15–20 cm depth. Forty randomly selected mesocosms were transferred from the NW to the SW site. The remaining 40 mesocosms were transferred within the NW site at the same elevation, representing *in situ* controls. After transfer, all plant-soil mesocosms were irrigated with 500 ml water within two hours (corresponding to a typical summer precipitation event of 23.7 l m^−2^) to avoid drying or death of enclosed beech seedlings after transfer.

After a pre-incubation period of 11 months for acclimatization, eight intact mesocosms were sampled at both NW and SW on 22^nd^ June 2011 (Sampling T1, representing ambient climate change). In order to simulate intensive summer drought, a translucent rain sheltering roof was established at SW from 27^th^ June 2011 to 9^th^ August 2011. Trenches around the 1 m tall rain shelter avoided influence of slope water. After 39 days of enhanced drought at SW, eight mesocosms from both NW and SW were sampled on 2^nd^ August 2011 (Sampling T2, representing ambient/intensive summer drought). For simulation of a severe precipitation event, the remaining plant-soil mesocosms of both sites were irrigated three times with 280 ml water each within two hours on 5^th^ August 2011. On 6^th^ August and 8^th^ August 2011, respectively, eight mesocosms were sampled at both NW and SW (Sampling T3 and T4, representing intensive precipitation). After deconstruction of the rainout shelter at SW on 9^th^ August 2011, the remaining mesocosms were left under ambient climate conditions at NW and SW, respectively, until the final sampling on 27^th^ September 2011 (Sampling T5).

### Sampling of the plant-soil mesocosms

For each sampling time, eight plant-soil mesocosms at both NW and SW were taken and treated as independent replicates. The intact mesocosms were carefully excavated by hand and processed within two hours after excavation. The beech seedlings were cut and separated into leaves and stems. The remaining soil-root mesocosms were manually separated into soil, gravel, dead organic material and living roots, which were washed with tap water to remove adhering soil. The plant parts were dried at 60°C for 48 hours for determination of dry biomass, which was significantly reduced at SW compared to NW for both above- and belowground plant biomass (Table S1).

The remaining mesocosm soil was homogenized by manual mixing for 10 min. A soil subsample was immediately frozen on dry ice and stored at −80°C for DNA/RNA extraction. A second soil subsample was freeze-dried for determination of carbon and nitrogen content. A third subsample of soil (100 g fresh weight) was immediately extracted 1:1.5 (soil:solution) with 0.5 M K_2_SO_4_ on a rotary shaker for one hour. Afterwards, extracts were vacuum filtered using pumps and glass fibre filters [2] and stored at −20°C for analysis of extractable soil C and N pools.

### Total soil carbon and nitrogen content

For analysis of soil C and N content, the freeze-dried soil samples were ball-milled (Retsch MM2, Retsch GmbH, Haan, Germany), weighed into tin capsules (1.5 mg soil per capsule) and analyzed in duplicates using an Elemental Analyzer ‘Euro-EA’ (Eurovector, Milano, Italy).

### Extractable soil carbon and nitrogen pools

Extractable soil C and N pools were analyzed as described by Dannenmann et al. [2]. For determination of ammonium and nitrate concentrations, a subsample of the filtered extracts was analyzed colorimetrically by a commercial laboratory (Dr. Janssen, Gillersheim, Germany). Total organic carbon (TOC) and total nitrogen (TN) in the extracts was quantified on an Infrared TOC analyzer with a coupled chemoluminescence-based total N module (DIMATEC GmbH, Germany). TOC concentrations of extracts were referred to as extractable dissolved organic carbon (DOC), while extractable dissolved organic nitrogen (DON) was calculated as the difference between TN and dissolved inorganic N (ammonium plus nitrate).

### Nucleic acid extraction

DNA and RNA from 0.4 g homogenized soil were co-extracted using the protocol described by Lueders [32] and the Precellys24 Instrument (PeqLab, Erlangen, Germany). Quality and quantity of the extracted nucleic acids were checked with a spectrophotometer (Nanodrop, PeqLab, Erlangen, Germany) and gel electrophoresis. Afterwards, the extract was divided into two subsamples. One was used for DNA analysis without further treatment. The second subsample was used to prepare RNA by digestion of co-extracted DNA with RNase free DNase I (Promega, Mannheim, Germany) according to manufacturer's instructions and subsequent purification using 3 M RNase free sodium acetate (pH 5.2) and isopropanol. Quality and quantity of the extracted RNA were checked with a spectrophotometer (Nanodrop, PeqLab, Erlangen, Germany) and gel electrophoresis. The absence of DNA was confirmed by performing PCR targeting the 16S rRNA gene using universal primers 968f (5′- AAC GCG AAG AAC CTT AC -3′) and 1401r (5′- CGG TGT GTA CAA GAC CC -3′) and the amplification protocol described by Schreiner et al. [33]. Afterwards, cDNA was synthesized using the ‘High capacity cDNA reverse transcription kit’ (Life Technologies, Darmstadt, Germany) according to manufacturer's instructions. The success of cDNA synthesis was confirmed by performing PCR targeting the 16S rRNA gene as described above. Both DNA and cDNA extracts were stored at −20°C until use.

### Real-time PCR assay

Quantitative real-time PCR (qPCR) was performed using an ABI 7300 Cycler (Life Technologies, Darmstadt, Germany) with the following assay reagents: dimethyl sulfoxid (DMSO) and bovine serum albumin (BSA) (Sigma, Germany), primers listed in Table 1 (Metabion, Martinsried, Germany) and 2x Power SYBR Green master mix (Life Technologies, Darmstadt, Germany). The respective reaction mixtures (total volume 25 µl) for quantification of the genes listed in Table 1 consisted of: 12.5 µl SYBR Green master mix, 5 pmol of each primer (for *apr* gene: 10 pmol of each primer), 0.5 µl 3% BSA and 2 µl DNA template. For the amplification of *nirS* and *nirK* genes, 0.5 µl DMSO was added additionally.

**Table 1:** Thermal profiles and primer used for real-time PCR quantification of different functional genes and transcripts.

Target gene	Source of standard	Primer	References	Thermal profile	No. of cycles
*nifH*	*Azospirillum irakense*	nifH-f, nifH-r	[63]	95°C-45s/55°C-45s/72°C-45s	40
*amoA* AOA	*Nitrosomonas europaea*	amo19F, CrenamoA16r48x	[49], [64]	94°C-45s/55°C-45s/72°C-45s	40
*amoA* AOB	Fosmid clone 54d9	amoA1F, amoA2R	[65]	94°C-45s/59°C-45s/72°C-45s	40
*nirS*	*Pseudomonas stutzeri*	cd3aF, R3cd	[66], [67]	95°C-45s/57°C-45s/72°C-45s	40
*nirK*	*Azospirillum irakense*	nirK876, nirK5R	[68], [69]	95°C-15s/63-58°C-30s/72°C-30s	5 a
				95°C-15s/58°C-30s/72°C-30s	40
*cnor*	*Sinorhizobium meliloti*	cnorB2f, cnorB6r	[20]	95°C-15s/60-55°C-30s/72°C-30s	5 a
				95°C-15s/55°C-30s/72°C-30s	40
*nosZ*	*Pseudomonas stutzeri*	nosZ2F, nosZ2R	[70]	95°C-15s/65-60°C-30s/72°C-30s	5 a
				95°C-15s/60°C-30s/72°C-30s	40
*chiA*	*Streptomyces griseus*	chiF2, chiR	[71]	95°C-30s/60°C-30s/72°C-60s	40
*apr*	*Pseudomonas aeruginosa*	FPapr1, RPapr2	[72]	95°C-20s/53°C-30s/72°C-60s	40

a Touchdown: −1°C per cycle.

For quantification, standard curves were calculated using serial dilutions (10^1^ to 10^6^ gene copies μl^−1^) of plasmid DNA containing PCR products of the respective genes listed in Table 1. PCR detection limit was assessed to 1 gene copy μl^−1^. In order to prevent PCR inhibition, the optimal dilution for each amplification assay was determined by dilution series of randomly chosen DNA and cDNA extracts in advance (data not shown). The qPCR assays were performed in 96-well plates (Life Technologies, Darmstadt, Germany) for all target genes as described in Table 1. All PCR runs started with a hot start at 95°C for 10 minutes. After each run, the specifity of the SYBR Green-quantified amplicons was checked by melting curve analysis and gel electrophoresis. The amplification efficiency was calculated from the formula Eff  =  [10^(−1/slope)^−1] and resulted in the following average efficiencies (standard deviation less than 5% of mean): *nifH*, 86%, *chiA*, 88%, *apr*, 89%, *amoA* ammonium-oxidizing archaea (AOA), 94%, *amoA* ammonium-oxidizing bacteria (AOB), 91%, *nirK*, 98%, *nirS*, 91%, *cnor*, 93%, and *nosZ*, 89%.

### Statistical analysis

Statistical analysis was performed using SPSS 11.5 (SPSS, Inc.). Data was evaluated by multivariate analysis of variance (ANOVA) at the significance level P <0.05. The normal distribution of the data was checked by the Kolmogorov-Smirnov test and histograms. If necessary, the data was log-transformed prior to analysis. The homogeneity of the variances was checked by the Levene test. For the pairwise comparison of means with the ANOVA, either the Tukey test or, if the homogeneity of the variances was not given, the Games-Howell test was used. In order to test significant effects between the sites at each time point, Student's T-test for independent samples was performed.

## Results

### Soil moisture

Mean values of gravimetric soil water content normalized to WHC are presented in Table 2. As expected, transferring plant-soil mesocosms from NW to SW resulted in a decrease in soil moisture from 63.5% WHC to 54.4% WHC after 11 months of acclimatization (T1). This reduced soil water content at SW compared to NW was observed during the whole sampling period and was especially pronounced at T2, where roof-intensified drought at SW reduced soil moisture to 44.5% WHC, while soil moisture at NW was not significantly changed by ambient summer drought (57.2% WHC). Simulated precipitation increased the soil water content to 62.3% WHC (NW, P > 0.05) and 53.0% WHC (SW), respectively. With ongoing season, soil moisture remained constant at both sites (on average 60.6% WHC at NW and 49.8% WHC at SW).

**Table 2:** Gravimetric soil moisture related to water holding capacity (WHC), total N and C contents as well as extractable N and C pools of soil of soils at NW and SW, sampled in June (T1), after 39 days drought in August (T2), 24 and 72 hours after rewetting in August (T3, T4) and in September (T5) (n = 8, standard deviation of the mean in parentheses).

	NW					SW					P _site_	P_ time_
	T1	T2	T3	T4	T5	T1	T2	T3	T4	T5		
Water content	63.5 (4.0) *	57.2 (8.6) *	62.3 (7.0) *	61.0 (11.0) *	60.3 (10.4) *	54.4 (7.0) *	44.5 (6.4) *	53.0 (9.1) *	48.8 (6.5) *	50.7 (10.6) *	**0.000**	**0.021**
% WHC	a	a	a	a	a	a	b	a	ab	ab		
N total	4.6 (0.3)	4.3 (0.7)	4.7 (0.8)	4.3 (1.0)	4.7 (1.0)	4.4 (0.7)	4.8 (1.0)	5.0 (0.8)	4.1 (1.1)	4.8 (0.9)	0.643	0.216
mg g^−1^ sdw	a	a	a	a	a	a	a	a	a	a		
NH4^+^	3.8 (1.8)	3.3 (1.1)	5.9 (1.0)	3.9 (0.8)	3.2 (1.6)	3.5 (2.2)	3.6 (1.0)	6.0 (1.1)	3.8 (1.5)	3.1 (1.0)	0.964	**0.000**
μg g^−1^ sdw	a	a	b	a	a	a	a	b	a	a		
NO3^−^	4.5 (2.5)	4.2 (2.5)	3.1 (1.3)	5.0 (2.8)	0.3 (0.1)	4.1 (2.9)	4.3 (2.3)	3.1 (0.9)	5.1 (2.1)	0.5 (0.3)	0.976	**0.000**
μg g^−1^ sdw	a	a	ab	a	b	ab	ab	ab	a	b		
DON	9.1 (3.5)	1.0 (1.5) *	2.3 (2.2)	3.3 (2.5)	5.9 (1.3)	9.8 (3.6)	5.4 (3.0) *	5.0 (3.6)	3.4 (1.8)	3.8 (2.9)	0.063	**0.000**
μg g^−1^ sdw	a	b	bc	bc	c	a	ab	ab	b	b		
C total	58.6 (4.3)	57.4 (9.6)	60.7 (10.7)	58.5 (15.8)	64.6 (16.6)	58.7 (10.8)	60.7 (13.1)	66.8 (8.2)	59.6 (13.5)	67.2 (11.8)	0.322	0.305
mg g^−1^ sdw	a	a	a	a	a	a	a	a	a	a		
DOC	72.2 (11.5)	20.5 (7.1)	51.5 (14.3)	48.9 (17.2)	52.2 (11.5)	71.2 (13.8)	23.6 (5.3)	55.7 (12.7)	57.4 (25.9)	45.2 (20.6)	0.654	**0.000**
μg g^−1^ sdw	a	b	c	c	c	a	b	ac	ac	bc		

Asterisks indicate significant differences between NW and SW at the respective sampling times (Student's T test), whereas lower case letters indicate differences among the sampling period for the respective site (multivariate ANOVA). Significant differences between the factors site and sampling time calculated by multivariate ANOVA are indicated by P values <0.05 (bold letters).

### Soil biochemical parameters

As expected, neither total soil N nor C contents differed between NW and SW or during sampling times and were 4.6 mg N g^−1^ soil dry weight (sdw) and 61.3 mg C g^−1^ sdw in average. Contrary, the measured labile pools (ammonium, nitrate, DON and DOC) were highly influenced by drought and rewetting with similar intensities and trends at NW and SW (except for DON). Therefore, data of NW at the respective time points were described as example in this result section (except for DON). Ammonium contents increased from 3.8 µg N g^−1^ sdw (NW, T1) to 5.9 µg N g^−1^ sdw (NW, T3) and declined again to 3.2 µg N g^−1^ sdw (NW, T5). Nitrate contents decreased from 4.5 µg N g^−1^ sdw (NW, T1) to 3.1 µg N g^−1^ sdw (NW, T3, P > 0.05), increased to 5.0 µg g^−1^ sdw (NW, T4, P > 0.05) and decreased to 0.3 µg N g^−1^ sdw (NW, T5). DON contents were similar between NW and SW at T1 (9.1 µg N g^−1^ sdw respective 9.8 µg N g^−1^ sdw), but while DON at SW declined continuously during sampling time to 3.8 µg g^−1^ sdw (T5), at NW the lowest DON value was observed at T2 (1.0 µg g^−1^ sdw) followed by a continuously increase to 5.9 µg g^−1^ sdw (T5). For DOC contents no difference between NW and SW was observed: DOC decreased from 72.2 µg C g^−1^ sdw (NW, T1) to 20.5 µg C g^−1^ sdw (NW, T2), increased after rewetting 51.5 µg C g^−1^ sdw (NW, T3) and remained constant hereafter. All data regarding soil biochemical parameters is summarized in Table 2.

### Abundance of functional transcripts involved in nitrogen cycling

**Figure 1:**
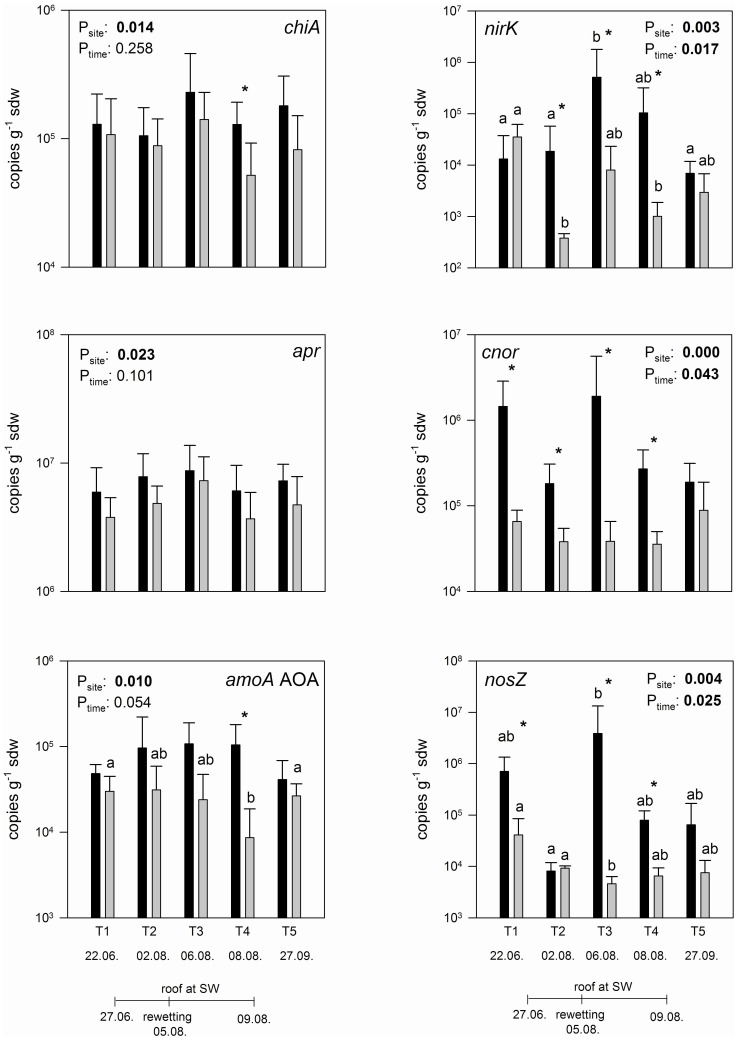
Transcript abundance of functional genes involved in the nitrogen cycle (*chiA*, *apr*, *amoA* AOA, *nirK*, *cnor* and *nosZ*) are shown for NW (black bar) and SW (grey bar) in June (T1), after 39 days drought in August (T2), 24 and 72 hours after rewetting in August (T3, T4) and in September (T5) (n = 8, error bars represent standard deviation of the mean). Asterisks indicate significant differences between NW and SW at the respective sampling times (Student's T test), whereas lower case letters indicate differences among the sampling period for the respective site (multivariate ANOVA). Significant differences between the factors site and sampling time calculated by multivariate ANOVA are indicated by P values <0.05 (bold letters).

In general, the genetic potential (based on the abundance levels of the investigated functional groups) was not or not consistently affected by the investigated climate change scenario (Fig. S1). In contrast, transcription pattern showed a significant site effect for all quantified genes with lower transcript numbers at SW compared to NW in average (Fig. 1). Transcripts of *nifH, amoA* AOB and *nirS* genes were undetectable in all samples.

Concerning mineralization, transcript numbers of *chiA* were within the range of 10^5^ transcripts g^−1^ sdw in average and thus 50 times lower compared to *apr* transcript abundance. Independent from the site, both *chiA* and *apr* transcript numbers were highest 24 hours after the simulated intense precipitation event (T3), with 2.3×10^5^ transcripts g^−1^ sdw (NW) and 1.4×10^5^ transcripts g^−1^ sdw (SW) for *chiA* respective 8.7×10^6^ transcripts g^−1^ sdw (NW) and 7.3×10^6^ transcripts g^−1^ sdw (SW) for *apr*. However, due to high variations among the replicates the increase was not significant.

In terms of ammonia oxidation, *amoA* transcripts of AOA dominated clearly over AOB, which were undetectable in all samples. On NW, AOA transcript numbers remained constant over time (7.9×10^4^ transcripts g^−1^ sdw in average), while at SW 72 hours after rewetting (T4) a significant but temporarily decrease from 3.1×10^4^ to 8.6×10^3^ transcripts g^−1^ sdw was observed, followed by an increase to 2.6×10^4^ transcripts g^−1^ sdw (T5).

Regarding denitrification, transcripts of the functional genes *nirK, nirS, cnor* and *nosZ* were quantified, targeting the stepwise reduction of nitrite (NO_2_^−^) to dinitrogen via nitric oxide (NO) and nitrous oxide (N_2_O). While *nirS* transcripts were undetectable in all samples, functionally redundant *nirK* transcript numbers ranged from 3.8×10^2^ to 5.1×10^5^ transcripts g^−1^ sdw and were highly affected by site, drought and simulated precipitation. At NW, *nirK* transcripts were highest 24 hours after rewetting (5.1×10^5^ transcripts g^−1^ sdw at T3) and lowest at T5 with 6.9×10^3^ transcripts g^−1^ sdw. In contrast, *nirK* transcript numbers at SW were severely decreased by roof-intensified drought at T2 (3.8×10^2^ transcripts g^−1^ sdw). Although rewetting led to an temporarily increase in *nirK* abundance up to 8.0×10^3^ transcripts g^−1^ sdw (T3, P > 0.05), transcripts decreased afterwards and remained lower hereafter compared to T1 at SW. However, transcript numbers of *nirK* were significantly reduced at SW compared to NW at the drought and rewetting treatment (T2–T4). In contrast to *nirK, cnor* transcripts at NW were more affected by drought and rewetting than at SW. At NW, a decrease of *cnor* transcript numbers from 1.5×10^6^ to 1.8×10^5^ transcripts g^−1^ sdw at T2 (drought) was observed (P > 0.05), followed by a temporarily increase to 1.9×10^6^ transcripts g^−1^ sdw after rewetting (P > 0.05). At SW, *cnor* decreased from 6.6×10^4^ transcripts g^−1^ sdw (T1) to 3.8×10^4^ transcripts g^−1^ sdw (T2, P > 0.05), remained constant during rewetting and increased to 8.9×10^4^ transcripts g^−1^ sdw at T5 (P > 0.05). Although the time-dependent effects were not significant, *cnor* transcript numbers were significantly lower at SW compared to NW for T1-T4. For *nosZ* transcripts, similar time patterns as for *cnor* were observed. Transcription levels of *nosZ* at NW decreased during drought to 8.1×10^3^ transcripts g^−1^ sdw (P > 0.05), increased significantly after rewetting (3.9×10^6^ transcripts g^−1^ sdw at T3) and declined again to 6.4×10^4^ transcripts g^−1^ sdw at T5 (P > 0.05). At SW, *nosZ* transcripts were highest at T1 (4.1×10^4^ transcripts g^−1^ sdw), declined to 4.6×10^3^ transcripts g^−1^ sdw after rewetting (T3) and increased again to 7.6×10^3^ transcripts g^−1^ sdw at T5 (P > 0.05). However, also *nosZ* transcripts were lower at SW compared to NW, except after drought (T2) and in September (T5).

## Discussion

### Biological nitrogen fixation is negligible in the investigated beech forest ecosystem

Despite general N limitation of forest ecosystems and the presence of *nifH* genes in all of our samples (2×10^7^ to 5×10^7^ copies g^−1^ sdw, Fig S1), no *nifH* transcripts were detected, indicating a negligible role of biological nitrogen fixation (BNF) in the investigated ecosystem. This is in contrast to other studies showing abundant BNF in temperate and boreal forests [34], [35], [36]. However, it might be possible that atmospheric N deposition in this study was sufficient to outbalance ecosystem N losses by gaseous emissions and leaching, thus providing no advantage in the highly energy demanding N_2_ fixation [37].

### Activity of bacteria involved in N mineralization

In terrestrial ecosystems, the majority of soil N is present in organic macromolecules like chitin, proteins and nucleic acids [22], [38], which cannot be assimilated by plants directly [39]. Therefore, abundances of *apr* and *chiA* transcripts were used as proxy for soil N mineralizing microbes [24], [40], [41]. For both *apr* and *chiA* transcript numbers a significant site effect was observed with lower transcripts under climate change conditions (increased soil temperature and decreased water availability) in average. This was surprising, as soil warming was shown to enhance biological processes like decomposition and N mineralization in general [42], [43]. However, reduced soil moisture may result in decreased microbial activity due to osmotic regulation (accumulation of compatible solutes in microbial cells), limited diffusive transport of substrates and extracellular enzymes and lower microbe motility [9]. In the investigated ecosystem, the negative effect of low water availability seamed to dominate over temperature-induced higher activity of microorganisms. This is in line with previous studies showing that net N mineralization rate was more limited by low soil moisture than promoted by increased temperature [44], [45]. Surprisingly, transcript levels for both *apr* and *chiA* remained unaffected by drought and rewetting at both NW and SW, which is in contrast to other studies showing rapidly increasing N mineralization after rewetting of dry soil due to accumulated plant and microbial necromass and microbial cell lysis caused by osmotic stress [15], [46].

It has to be taken into account that transcript abundances of *chiA* and *apr* must not necessarily reflect *in situ* mineralization rates because of post-transcriptional and –translational modifications of the underlying mRNA and/or enzymes [47]. Moreover, besides chitinases and proteases a lot of other enzymes contribute to N mineralization in soil. When relating total mineralization rates to abundance and activity of microbes the role of fungal proteases has to be considered especially in forest soils. However, here the molecular basis is fairly unknown so far, as sequenced fungal isolates are rare.

### Activity pattern of ammonia oxidizers

Nitrification, the stepwise oxidation of ammonia to nitrate via nitrite, was quantified via expression of archaeal and bacterial *amoA* genes. Abundance of AOA ranged from 4×10^7^ to 2×10^8^ copies g^−1^ sdw and was thus 100-times higher than AOB abundance (Fig S1). Consequently, on transcript level AOB was undetectable. This numerical dominance of AOA over AOB was found in several studies [19], [48], [49]. Although nitrification as aerobic process could be expected to be positively influenced by climate change (increased soil temperature and reduced soil moisture and thus higher oxygen content) as reported previously [50], [51], our results revealed lower AOA transcript numbers at SW compared to NW in average. It is likely that this was due to a soil moisture threshold for optimal nitrification, which is in the range of 60–65% of WHC at the investigated study site [3]. While water availability at NW was within this range, soil moisture at SW was reduced far beyond this optimum. Surprisingly, although AOA were thought to be highly responsive to changing environmental conditions and more dynamic than AOB [19], no drought or rewetting effect on AOA transcript abundance was observed in this study. This was also reflected in soil ammonium concentrations which remained constant during the sampling period, with the exception of temporarily increased ammonium levels after rewetting. As archaeal *amoA* transcripts were not reduced, this might be due to decreased expression of bacterial *amoA* genes because of lower oxygen availability (which could not be measured as AOB were below detection limit) and/or increased mineralization as described above. However, ammonium concentrations declined within 72 hours after rewetting, indicating high ammonium immobilization by heterotrophs and/or rapid plant uptake.

### Transcript abundances of different genes involved in denitrification related processes

The nitrate produced during nitrification may serve as substrate for denitrification, resulting in the stepwise reduction to gaseous compounds (NO, N_2_O, N_2_) and consequently in a loss of N from the ecosystem. In the present study, the last three steps of denitrification (the reduction of NO_2_^−^, NO and N_2_O) were investigated by quantifying the transcripts of *nirK/nirS*, *cnor* and *nosZ* genes, respectively. Denitrification is closely linked to labile C and nitrate availability as well as oxygen partial pressure [52] and thus can be assumed to be highly sensitive to climate change with increased temperature and fluctuating water regimes.

The reduction of NO_2_^−^ is catalyzed by two functionally redundant enzymes encoded by *nirK* and *nirS* genes which do never co-occur in one organism [53]. The numerical dominance of *nirK* over *nirS* gene abundance observed in this study (Fig S1) was reported previously in soil ecosystems [41], [54], [55]. However, the low *nirS* gene abundance in our samples resulted in undetectable transcript numbers of *nirS*. Interestingly, transfer from NW to SW representing ambient climate change did not decrease *nirK* transcripts. This might be explained by enhanced microbial activity because of increased temperature which outbalanced the negative effect of higher soil oxygen content [3] and/or the presence of anaerobic microsites due to elevated soil respiration and clayey texture promoting denitrification [56], [57]. This would be in agreement with the observation that *nirK* was not decreased during ambient summer drought at NW. In contrast, roof-intensified drought at SW resulted in a dramatic decline of *nirK* transcripts, indicating that soil moisture and consequently substrate diffusion and presence of anaerobic microsites were decreased to an extent that could not be compensated by higher temperature. However, rewetting led to a rapid increase of *nirK* transcript numbers on both sites, which resulted consequently in reduced soil nitrate concentrations. This rapid recovery from drought stress after rewetting was reported previously for *nirK* harbouring microorganisms [19].

In contrast to *nirK*, *cnor* and *nosZ* transcripts were already influenced by ambient climate change conditions, resulting in decreased transcript levels of both genes at SW compared to NW at T1. Denitrifiers are heterotrophic organisms and among others dependent on labile C sources [52]. As plant biomass was significantly reduced at SW, it is likely that also root exudation and consequently labile C compounds were lowered [2], resulting in decreased denitrification activity. On the other hand, the different response might be due to higher oxygen sensitivity of *cnor* and *nosZ* compared to *nirK*, which at least is known for *nosZ* [58], [59]. Accordingly, after ambient summer drought at NW as well as after roof-intensified drought at SW both *cnor* and *nosZ* transcripts showed decreasing trends, but with different intensities. Interestingly, after rewetting a relative increase of *cnor* and *nosZ* was only observed at NW, although nitrate concentrations and DOC availability were similar at both sites. It might be speculated that roof-intensified drought at SW reduced soil moisture far beyond the optimum threshold for NO_2_^−^ and N_2_O reduction, thus causing severe environmental stress [60]. Consequently *cnor* and *nosZ* harboring microorganisms need time for recovery when environmental conditions become more favorable again. Such resilience according to changing moisture systems was observed previously for *nosZ* harboring microorganisms.[61] On the other hand, rewetting of dry soil might have caused increased cell lysis due to high osmotic stress [16], which would also explain the lack of immediate response.

### Synthesis

**Figure 2:**
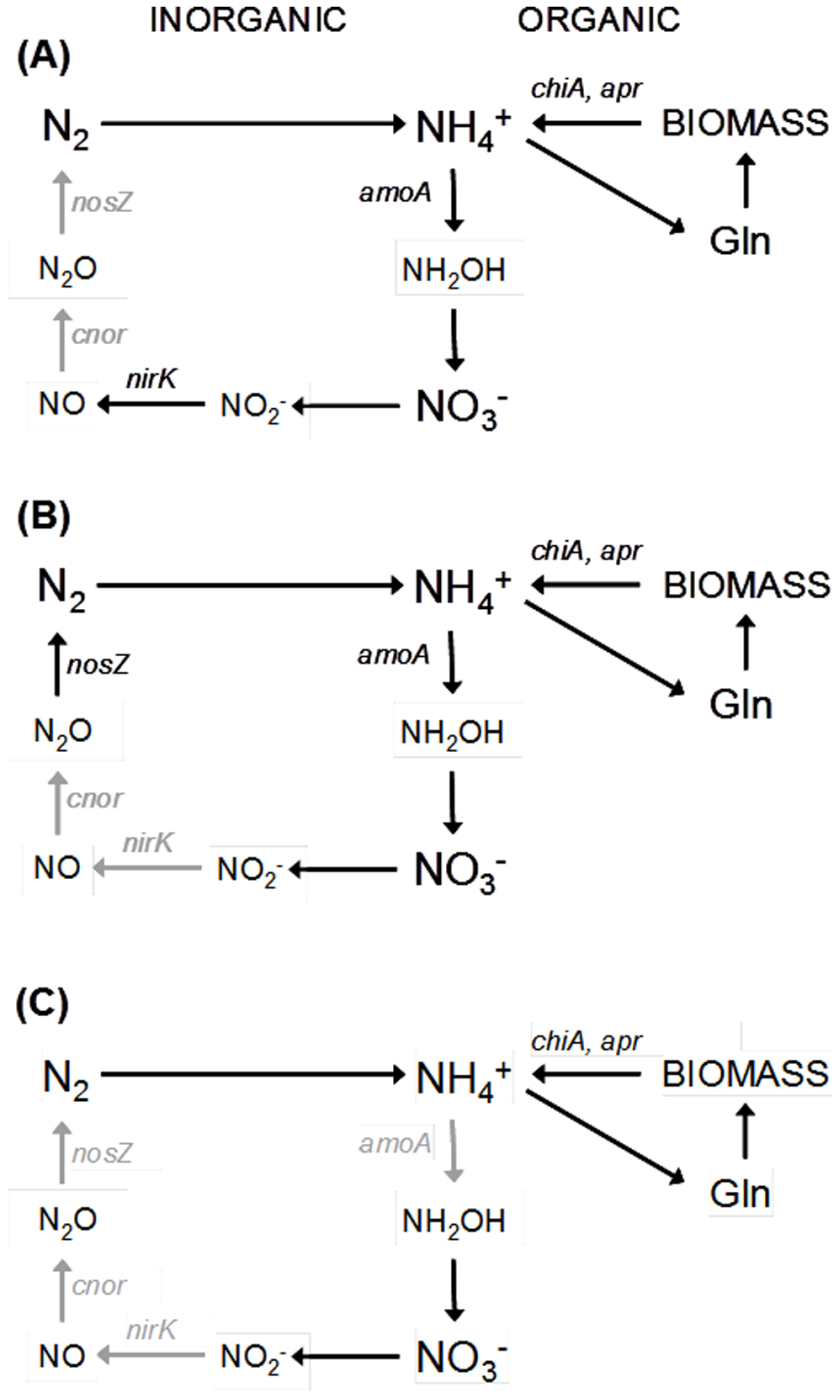
Scheme of the microbial nitrogen cycle under different climate change conditions. (A) comparison between NW and SW at ambient climate change (T1), (B) comparison between NW and SW at ambient/roof-intensified summer drought (T2) and (C) comparison between NW and SW after rewetting (T3, T4). Decreased N turnover processes under climate change indicated by significantly lower transcripts at SW compared to NW are shown in grey (P <0.05).

In conclusion, climate change conditions including higher temperature and lower soil moisture resulted in significant site effects with decreased expression of investigated functional genes at SW while the genetic potential was not or not consistently affected. Our data revealed that particularly transcripts related to denitrification were affected by climate change. Already transfer from NW to SW without further treatment resulted in significant reduction of *cnor* and *nosZ* transcripts while *nirK* remained unaffected (Fig. 2A), suggesting the possibility of higher NO losses at moderately increased soil temperatures and decreased water availability. Besides its potential as greenhouse gas, NO is an important signaling molecule for plants participating in a variety of physiological processes including seed germination, root formation, programmed cell death as well as defense and stress responses [62]. Therefore, a possible increase in NO production under climate change may also directly affect plant performance in near future. Severe drought decreased additionally *nirK* transcripts (Fig. 2B), which could indicate N_2_ as main gaseous denitrification product at SW. This is in contrast to ambient summer drought at NW, where decreasing trends for *cnor* and *nosZ* transcripts were observed while *nirK* remained unaffected. This is similar to the activity pattern at SW in June (T1) and could suggest higher gaseous NO losses at NW after natural summer drought. Although all investigated denitrification transcripts were significantly lower at SW compared to NW after rewetting (Fig. 2C), *nirK* increased, *cnor* remained constant and *nosZ* decreased relative to the abundance at severe drought, suggesting possible higher gaseous NO and N_2_O losses. In contrast, rewetting resulted in an increase of all denitrification transcripts at NW. Taken all together, our data suggest that climate change could increase the emission of greenhouse gases (NO, N_2_O), but this effect might be only temporarily as in September no difference between the denitrification transcript numbers at NW and SW was observed.

As *amoA* AOA transcripts were reduced after rewetting at SW but *chiA* and *apr* transcripts were not affected (Fig. 2C), this suggests that climate change triggered a truncation of soil microbial N cycling in this ecosystem type with decreasing importance of microbial nitrate production and consumption, whereas the effect on N mineralization was not thus pronounced.

Overall, the observed shifts in N turnover appeared to be more related to altered transcription patterns due to environmental factors than to changes in the genetic potential of the microbial community. Accordingly, transcript levels recovered at the end of the growing season under climate change conditions, indicating resilience of the respective microorganisms.

Although our study provides evidence that analysis of N cycle transcript abundances is a useful tool to reconstruct the microbial N cycle and to analyze the response of N cycle processes to climate change at the level of genes encoding for single enzymatic steps, it has to be taken into account that transcript levels of functional genes may allow prediction but must not reflect actual turnover rates. Therefore, more long-term studies measuring gross/net N fluxes additionally to functional genes and their transcripts are required, which would offer the possibility to link biogeochemical quantification of gross N turnover with gene expression for single enzymatic steps. With respect to functional redundancy within microbial populations, also community structure should be investigated to further sharpen our understanding of climate change effects on resilience and vulnerability of N cycle processes.

## Supporting Information

Figure S1 **Copy numbers of functional genes involved in the nitrogen cycle (** ***nifH*** **,** ***chiA*** **,** ***apr*** **,** ***amoA*** **AOA,** ***amoA*** **AOB,** ***nirK*** **,** ***nirS*** **,** ***cnor*** **and** ***nosZ*** **) are shown for NW (black bar) and SW (grey bar) in June (T1), after 39 days drought in August (T2), 24 and 72 hours after rewetting in August (T3, T4) and in September (T5) (n = 8, error bars represent standard deviation of the mean).** Asterisks indicate significant differences between NW and SW at the respective sampling times, whereas lower case letters indicate differences among the sampling period for the respective site.
(PDF)

Table S1 **Dry plant biomass at NW and SW, sampled in June (T1), after 39 days drought in August (T2), 24 and 72 hours after rewetting in August (T3, T4) and in September (T5) (n = 8, standard deviation of the mean in parentheses).** Asterisks indicate significant differences between NW and SW at the respective sampling times (Student's T test), whereas lower case letters indicate differences among the sampling period for the respective site (multivariate ANOVA). Significant differences between the factors site and sampling time calculated by multivariate ANOVA are indicated by P values <0.05 (bold letters).
(PDF)
