# Correction: Climate Change Induces Shifts in Abundance and Activity Pattern of Bacteria and Archaea Catalyzing Major Transformation Steps in Nitrogen Turnover in a Soil from a Mid-European Beech Forest

**DOI:** 10.1371/journal.pone.0116614

**Published:** 2014-12-23

**Authors:** The PLOS ONE Staff

The third author’s name is spelled incorrectly. The correct name is: Carolin Bimüller. The fifth author’s name is spelled incorrectly. The correct name is: Ingrid Kögel-Knabner.

The correct citation is: Gschwendtner S, Tejedor J, Bimüller C, Dannenmann M, Kögel-Knabner I, et al. (2014) Climate Change Induces Shifts in Abundance and Activity Pattern of Bacteria and Archaea Catalyzing Major Transformation Steps in Nitrogen Turnover in a Soil from a Mid-European Beech Forest. PLoS ONE 9(12): e114278. doi:10.1371/journal.pone.0114278

Additionally, there is a missing affiliation for author Ingrid Kögel-Knabner. Ingrid Kögel-Knabner is also affiliated with: Institute for Advanced Study, Technische Universität München, Lichtenbergstraße 2a, D-85748 Garching, Germany.
