# Supplementary figures and images for: Climate Change Induces Shifts in Abundance and Activity Pattern of Bacteria and Archaea Catalyzing Major Transformation Steps in Nitrogen Turnover in a Soil from a Mid-European Beech Forest
Source: PLoS One. 2014 Dec 2;9(12):e114278. doi: 10.1371/journal.pone.0114278 (PMC4252137; doi:10.1371/journal.pone.0114278)

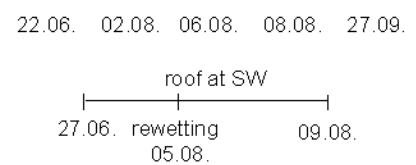

Supplement: Figure S1 — Copy numbers of functional genes involved in the nitrogen cycle ( nifH , chiA , apr , amoA AOA, amoA AOB, nirK , nirS , cnor and nosZ ) are shown for NW (black bar) and SW (grey bar) in June (T1), after 39 days drought in August (T2), 24 and 72 hours after rewetting in August (T3, T4) and in September (T5) (n = 8, error bars represent standard deviation of the mean). Asterisks indicate significant differences between NW and SW at the respective sampling times, whereas lower case letters indicate differences among the sampling period for the respective site. (PDF) [file pone.0114278.s001.pdf]
