# Supplementary material for: Climate Change Induces Shifts in Abundance and Activity Pattern of Bacteria and Archaea Catalyzing Major Transformation Steps in Nitrogen Turnover in a Soil from a Mid-European Beech Forest
Source: PLoS One. 2014 Dec 2;9(12):e114278. doi: 10.1371/journal.pone.0114278 (PMC4252137; doi:10.1371/journal.pone.0114278)
Supplement: Table S1 — Dry plant biomass at NW and SW, sampled in June (T1), after 39 days drought in August (T2), 24 and 72 hours after rewetting in August (T3, T4) and in September (T5) (n = 8, standard deviation of the mean in parentheses). Asterisks indicate significant differences between NW and SW at the respective sampling times (Student's T test), whereas lower case letters indicate differences among the sampling period for the respective site (multivariate ANOVA). Significant differences between the factors site and sampling time calculated by multivariate ANOVA are indicated by P values <0.05 (bold letters). (PDF) [file pone.0114278.s002.pdf]

Table S1

Dry plant biomass at NW and SW, sampled in June (T1), after 39 days drought in August (T2), 24 and 72 hours after rewetting in August (T3, T4) and in September (T5) (n=8, standard deviation of the mean in parentheses). Asterisks indicate significant differences between NW and SW at the respective sampling times (Student's T test), whereas lower case letters indicate differences among the sampling period for the respective site (multivariate ANOVA). Significant differences between the factors site and sampling time calculated by multivariate ANOVA are indicated by P values < 0.05 (bold letters).

	NW					SW					P_{site}	P_{time}
	T1	T2	T3	T4	T5	T1	T2	T3	T4	T5		
Belowground g dw	1.9 (0.8) * a	2.3 (1.4) * a	2.0 (0.7) a	2.1 (0.2) * a	2.3 (0.8) a	1.0 (0.3) * a	1.2 (0.2) * a	1.6 (0.8) a	1.5 (0.4) * a	1.6 (0.5) a	**0.000**	0.673
Aboveground g dw	2.6 (1.1) * a	3.1 (1.6) * a	2.7 (0.7) a	2.6 (0.4) a	2.0 (0.4) * a	1.5 (0.5) * a	1.8 (0.4) * a	2.2 (0.6) a	2.1 (0.8) a	0.6 (0.2) * b	**0.000**	0.157
